# Single-stage combined anterior retropharyngeal and posterior approach for the resection and reconstruction of C2 metastatic tumors: A case report

**DOI:** 10.3892/etm.2014.1493

**Published:** 2014-01-21

**Authors:** XIAN-FENG REN, YONG-MING XI, GUO-QING ZHANG, WEN-JIU YANG, XU ZHANG, DE-LING YANG, YOU-GU HU

**Affiliations:** Department of Orthopedic Surgery, The Affiliated Hospital of Medical College, Qingdao University, Qingdao, Shandong 266003, P.R. China

**Keywords:** upper cervical spine, surgery, surgical approach

## Abstract

This study reports the case of a 44-year-old male who had experienced severe neck pain for one month and was diagnosed with a metastatic tumor of the left C2 vertebral body and the left transverse process. The tumor was distributed to layers A–D and sectors 3–7 according to the Weinstein-Boriani-Biagini classification, and was in Category IV according to the Harrington classification system. A conventional posterior cervical approach was used to resect the left transverse process and part of the tumor in a piecemeal fashion, and spinal instrumentation was also performed. Gelfoam and absorbable hemostatic gauze were placed ventrally to the left vertebral artery and the left C3 nerve root over the tumor bed to prevent their accidental injury in the subsequent anterior approach. A high anterior retropharyngeal approach was then used to resect the tumorous C2 vertebral body by corpectomy and to perform anterior reconstruction. Six months after the surgery, the patient remained pain free. Therefore, C2 metastatic tumor resection and spinal reconstruction can be fulfilled by a single-stage combined high anterior retropharyngeal and posterior approach.

## Introduction

The upper cervical spine is an anatomically and biomechanically unique area. Metastatic involvement in this region is uncommon and few studies have been published to direct the treatment of it. Although the improvement in adjuvant therapy has led to a reduction in the number of patients requiring surgery for metastatic disease, surgery remains critical in the treatment of metastatic spinal tumors (1). The indications that surgical intervention of upper cervical lesions is required include evidence of gross instability due to fracture or bony destruction, gross malalignment, neurologic compromise due to malalignment or tumor compression, and a life expectancy of >3 months. Due to the close proximity to the neurovascular and soft tissues of the upper cervical spine, the resection and reconstruction of a metastatic tumor is challenging for spinal surgeons.

The majority of C2 metastatic tumors invade the vertebral body and the anterior approach often represents the most direct route to the lesion (2). Consequently, a transoral approach (3) or a transmandibular approach (4) is often employed, which provides direct access to the upper cervical region. However, the transoral approach has a high risk of infection, and it is difficult to perform the fixation extended to C3 due to the obstruction of the tongue and the jaw. The transmandibular approach is inappropriate for patients with a limited life expectancy due to the long time period required for bone healing. Furthermore, due to the limited life expectancy of patients with metastatic tumors, the main purpose of surgery is to improve life quality, and an approach with numerous complications and large trauma does not satisfy this requirement.

The high anterior retropharyngeal approach is an extension of surgical exposure to the lower cervical spine, allowing exposure from the ventral arch of C1 continuously to the lower cervical spine (5–7). It is entirely extraoral and extramucosal and is used for decompression of the spinal canal as well as for stabilization. The present study reports a successful outcome following a single-stage combined anterior retropharyngeal and posterior approach for resection of a C2 metastatic tumor and reconstruction of spinal stability.

## Case report

A 44-year-old male was admitted to the Affiliated Hospital of Medical College, Qingdao University (Qingdao, China) after experiencing severe neck pain for one month. The patient complained of intolerable pain in the occipitocervical area. The pain did not radiate to the upper extremities, and cervical motion was limited in all directions by pain and muscle spasm. Examination identified a spasmodic neck muscle and the neurological examination was normal. This study was conducted in accordance with the Declaration of Helsinki and with approval from the Ethics Committee of the Affiliated Hospital of Medical College, Qingdao University. Written informed consent was obtained from the participant.

**Figure 1:**
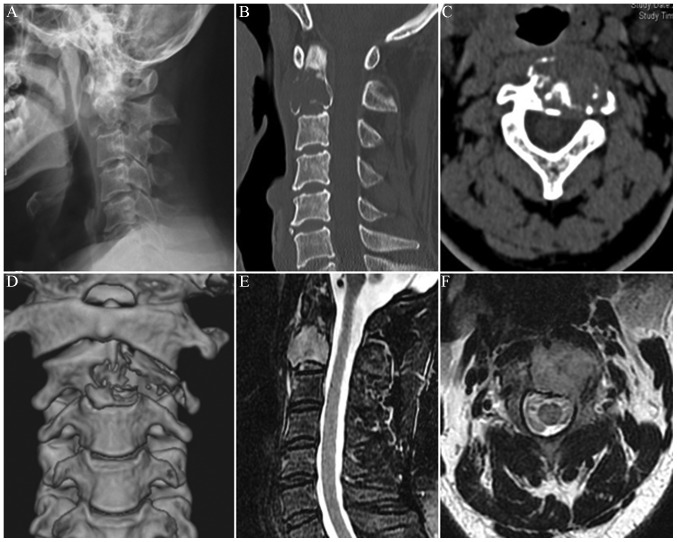
Series of images of the C2 metastasis. (A) Lateral X-ray shows a C2 destructive process, which was confirmed by (B and C) CT, (D) CT 3D and (E and F) MRI imaging. CT, computed tomography; 3D, three dimensional; MRI, magnetic resonance imaging.

**Figure 2:**
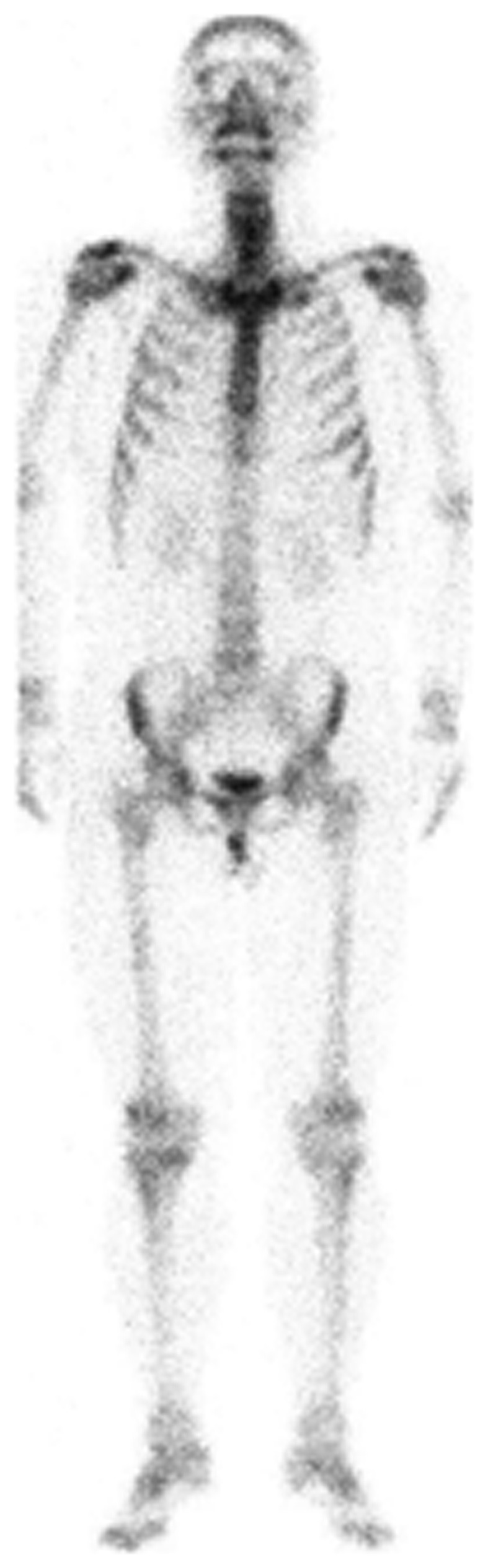
Bone scan showed diffuse increased uptake of the isotope at the level of the C2 vertebra, without other abnormality elsewhere in the skeleton.

**Figure 3:**
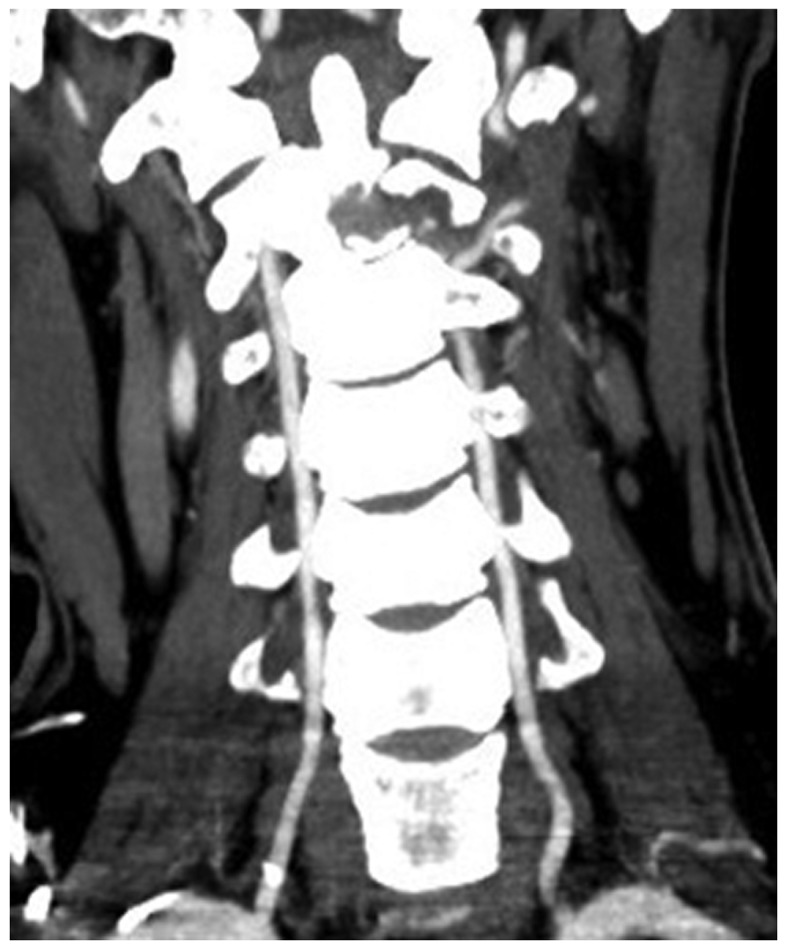
CT angiography showed destruction of the C2 body and that the VA was displaced. CT, computed tomography; VA, vertebral artery.

X-ray showed a poorly demarcated lesion of the C2 vertebral body (Fig. 1A). Computed tomography (CT) scanning and CT three-dimensional (3D) reconstruction confirmed an erosive lesion of the left C2 vertebral body and the left transverse process with surrounding soft tissue mass (Fig. 1B–D). Magnetic resonance imaging (MRI) showed that the lesion extended from the vertebral body to the neural foramen and the transverse process. The brainstem and the cervical spinal cord were not compressed (Fig. 1E and F). Bone scan demonstrated diffuse increased uptake of the isotope at the level of the C2 vertebra, without other abnormalities elsewhere in the skeleton (Fig. 2). CT angiography identified that the left vertebral artery (VA) was topically encapsulated by the tumor and displaced, but did not show any pathological vascularization (Fig. 3). Enhanced CT of the upper abdomen identified a lesion in the liver. The C2 metastatic tumor was distributed to layers A–D and sectors 3–7 according to the Weinstein-Boriani-Biagini classification (8), and was in Category IV according to the Harrington classification system (9).

Following consultation with the general surgeon and medical oncologist and extensive discussion with the patient and their family regarding the risks and benefits of surgery, the decision was made to perform the surgery by a combined anterior retropharyngeal and posterior cervical approach with somatosensory evoked potential monitoring. Following endotracheal intubation, the patient was positioned prone with skull traction to maintain spinal stability and a conventional posterior cervical approach was taken through a midline incision from the occiput to the C4 spinous process. Care was taken not to detach the paraspinal musculature from its insertion of the C2 spinal process. The left C2 facet and part of the left lateral C2 lamina were resected to decompress the C3 nerve root and to identify the destroyed left transverse process and the tumor surrounding it. The left transverse process and part of the tumor were resected in a piecemeal fashion to expose the left VA. Gelfoam and absorbable hemostatic gauze (Ethicon, San Lorenzo, PR, USA) were placed ventrally to the left VA and the left C3 nerve root over the tumor bed to prevent their accidental injury in the subsequent anterior approach. Posterior fixation was fulfilled by the placement of a polyaxial screw of an appropriate length into the right lateral mass of C1–4 and the left lateral mass of C1 and C4. The wound was closed in layers over a suction drain. Subsequently, the patient was turned to a supine position and a high anterior retropharyngeal approach was taken. The skin incision was made along the inferior edge of the mandible back to the ventral edge of the sternocleidomastoid muscle. The tumorous C2 vertebral body was resected by corpectomy. The edges of the tumor were identified and a intralesional extracapsulary resection was performed. A 12-mm-diameter titanium cage (Medtronic, Memphis, TN, USA) filled with polymethylmethacrylate cement was inserted in the space between the anterior arch of C1 and the upper endplate of the C3 vertebral body. A titanium cervical plate was then placed between the C1 anterior arch and the C3 vertebral body. The wound was closed in layers over a suction drain in the retropharyngeal space. The surgery time was 5 h, and the estimated blood loss was 1,000 ml.

**Figure 4:**
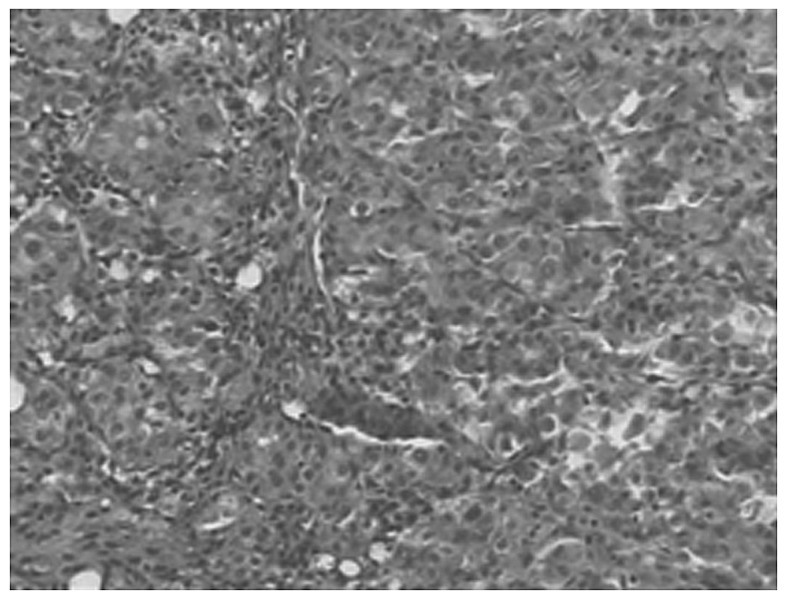
Postoperative pathological examination confirmed moderately differentiated hepatocellular liver cancer (hematoxylin-eosin; magnification, ×200).

**Figure 5:**
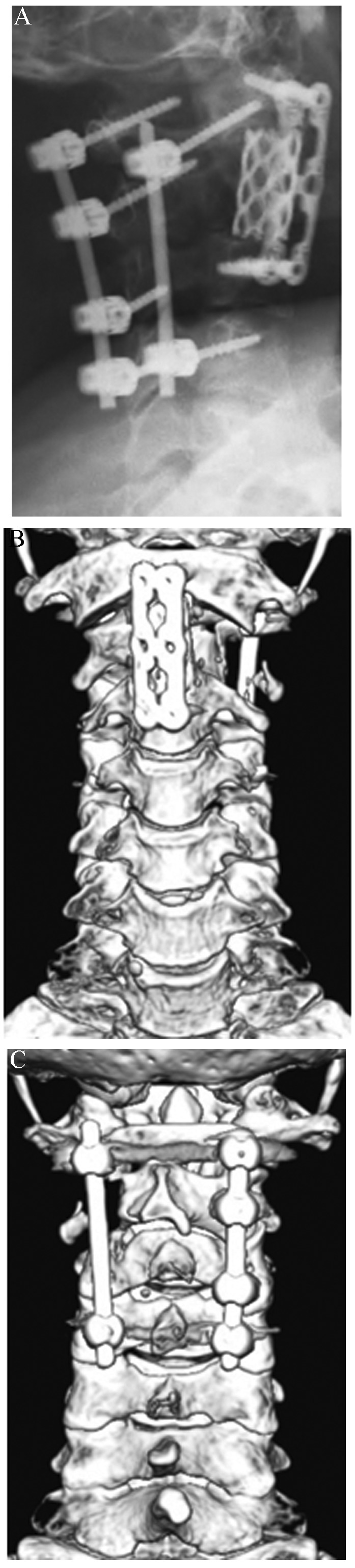
Postoperative images at the six month follow up showed no signs of implant dislocation. (A) Lateral X-ray. (B and C) CT 3D. CT, computed tomography; 3D, three dimensional.

Following the surgery, a Philadelphia collar was applied to the patient and the severe neck pain disappeared. Pathological examination confirmed moderately differentiated hepatocellular liver cancer (Fig. 4). Two weeks after the surgery, the patient received transcatheter arterial chemoembolization. The six-month follow-up X-ray (Fig. 5A) and CT 3D (Fig. 5B and C) showed no signs of implant dislocation and indicated persisting clinical success.

## Discussion

To the best of our knowledge, involvement of the upper cervical spine in metastases is uncommon and the literature review information regarding C2 metastasis consists of only a few case reports (14,17,18). Surgical intervention of C2 metastasis is a significant challenge for spinal surgeons.

Several approaches have been described for access to the upper cervical spine. The transoral approach, which provides the direct exposure for anterior decompression of the spinal cord and brainstem of the upper cervical spine, is most useful for resection of small, ventrally based tumors. However, it has a high risk of infection and it is difficult to perform the fixation extended to C3 due to the obstruction of the tongue and jaw (10). The transmandibular approach or its variations, which offer a wider exposure in the upper cervical region, are alternatives to the transoral route (3). However, it requires splitting of the mandible and thus is inappropriate for patients with limited life expectancy due to the long time period of bone healing required. Furthermore, several complications, such as velopharyngeal dysfunction, pharyngeal wound dehiscence, lingual neuropathy and cosmetic deformity have been reported in certain studies (11,12). The lateral retropharyngeal approach obtained with the retrovascular approach is not as direct as the anterior approach and it is difficult to perform long-segment anterior reconstruction (13). The posterior approach also has been reported to treat ventrally located upper cervical spine tumors, yet it does not provide adequate exposure of the tumor around the anterior midline. The high anterior retropharyngeal approach is an extension of the surgical exposure to the lower cervical spine, allowing exposure from the ventral arch of C1 continuously to the lower cervical spine. It has been demonstrated to be effective for C2 lesion resection and fixation to treat trauma, deformity and chronic inflammatory diseases. As far as C2 metastasis is concerned, few cases have been reported (14).

In the present study, the posterior approach was employed to resect part of the tumor and fulfill the posterior fixation to reinforce spinal stability during the position change of the patient. Subsequently the high anterior retropharyngeal approach was chosen to resect the ventrally located metastatic tumor and fulfill the reconstruction of the spinal alignment. It has been suggested that concomitant anterior and posterior fixation enhance the immediate stability of the spine (15). Preoperatively, it is important to evaluate the vascularity of the tumor and the association of the tumor mass with the vertebral arteries. As magnetic resonance angiography or CT angiography is less invasive and easier to conduct, digital subtraction angiography is rarely used to evaluate the vascularity of the lesion. Preoperative embolization is helpful in reducing intraoperative bleeding when an intralesional procedure is planned, but it is performed only rarely due to common vascularity with the cervical cord and should be only performed in experienced institutions (16). In the present study, CT angiography was used to evaluate the vascularity of the tumor and the association of the tumor mass with the vertebral arteries. There was no main arterial supply to the tumor, so preoperative embolization was not performed. During the surgery, Gelfoam and absorbable hemostatic gauze were placed ventrally to the left VA over the tumor bed to prevent accidental injury in the subsequent anterior approach.

The treatment of patients with metastatic disease of the cervical spine requires multidisciplinary cooperation between treatment team members, including a pathologist, medical and radiation oncologists and the spinal surgeon. In the present study, a combined posterior and high anterior retropharyngeal approach was used to resect a C2 metastatic tumor. This does not signify that other approaches are unsuitable for the resection and reconstruction of C2 metastasis; each case should be considered individually to determine the most appropriate surgical approach. If surgery is considered, the following factors should be taken into consideration when choosing the surgical approach: The experience of the surgeon, the life expectancy of the patient, the location, size and extent of the tumor, the stability of the spine and the neurological involvement.
